# A cross-sectional study on factors influencing patient participation in undergraduate medical education in a public and private hospital in Johannesburg, South Africa

**DOI:** 10.1186/s12909-023-04663-w

**Published:** 2023-09-21

**Authors:** Nicholas Makins, Tamiraa Naidoo, Taariq Hassim, Ohunayo Babalola, Charlize Dormehl, Remind Mkhabela, Lorenzo Degni, Kgotatso Liz Motloutsi, Mantoa Mokhachane

**Affiliations:** Unit for Undergraduate Medical Education, School of Clinical Medicine, University of the Witwatersrand, Johannesburg, South Africa

**Keywords:** Patient participation, Undergraduate medical education, Informed consent

## Abstract

**Background:**

The active involvement of patients in medical education is a common practice globally. Despite this, there is a global paucity of data on patients’ views on their role in medical education. As such this study aimed to identify factors that influence patient participation in undergraduate medical education in public and private hospitals in Johannesburg.

**Methods:**

A cross-sectional study was conducted, using a 23-question, self-designed, paper questionnaire to collect data on patients’ perceptions of student involvement in their care – with regard to consent, confidentiality, ethics, and patient preferences. Participants were recruited on a voluntary basis in the Departments of Medicine, Surgery, and Gynaecology, at selected hospital sites. Fisher’s Exact and Chi-Square statistical tests were used where appropriate.

**Results:**

Two hundred and one adult patients, comprised of 150 public sector patients and 51 private sector patients, completed the questionnaire. One hundred and sixty-nine patients (84,1%) were willing to participate in undergraduate medical education and no notable difference between these sectors was demonstrated (*p* = 0,41). The results further demonstrated that the main factors influencing patient participation in undergraduate medical education across both sectors were (1) the presence of a supervising professional, (2) the perceived degree of invasiveness of a procedure, and (3) the perceived expertise of the student. In addition, data across other key themes such as consent, confidentiality, ethics, and patient preferences and perceptions were elucidated.

**Conclusions:**

This study demonstrates that the majority of inpatients across the public and private sectors are willing to participate in undergraduate medical education to facilitate the development of healthcare professionals. It also demonstrated that most patients have a positive experience. However, more measures of quality informed consent need to be instituted to optimise the current role of the South African public health sector, whilst facilitating the development of a similar role for the South African private sector in future clinical education. In addition, further research is necessary to evaluate these findings in a South African context.

**Supplementary Information:**

The online version contains supplementary material available at 10.1186/s12909-023-04663-w.

## Background

The main goal of undergraduate medical education is to provide students with the necessary skills and knowledge, to deliver high-quality medical care [1]. To achieve this the Association of American Colleges and the United Kingdom General Medical Council recommend that medical students receive exposure to patients as early as possible during their training [2].

Whilst patients have always formed an essential component to medical education, their historical role has primarily been as passive aids to learning [3]. However, over the past two decades, the active involvement of patients as educators has increased to broaden curricula to include, promote and improve the psychosocial aspects of health, contextual learning, and patient-centred care. Despite the increasingly active involvement of patients in medical education there is a global paucity of data on patients’ views on their role in medical education, and little South African data around this topic exists [4].

South Africa is unique in that it does not have a universal healthcare system. Instead, the South African healthcare system has two parallel branches namely: the public healthcare system and the private healthcare system which co-exist and operate together [5]. The vast majority of the population (approximately 80%) make use of the public healthcare system that is subsidised by the South African government, via taxation. The remaining minority of the population (approximately 20%) make use of the private healthcare system in which they voluntarily make out-of-pocket contributions towards health insurance (colloquially known as medical aid schemes) in favour of receiving more timeous care in resource rich settings [5]. Private hospitals in South Africa only admit patients who have health insurance whilst patients without health insurance are admitted to public hospitals.

In contrast to the parallel systems of care that co-exist, the system of education and training of new healthcare professionals in South Africa is concentrated in the public health sector within ‘academic’ hospitals and few public–private partnerships for academic training exist [6]. This system of education and training introduces multiple areas of interest and consideration when students interact with patients for the purposes of learning.

### Ethico-legal considerations

There are multiple legal and ethical obligations that need to be abided by when teaching medical students with real patients [7]. In the South African contest, this includes university guidelines, professional body guidelines, and legislation on patient rights [7]. Undertaking an examination without complete consent impedes considerably on patient rights [7]. Despite this, international literature shows that these ethical and legal requirements are often neglected [8]. In addition, available literature describes inadequate consent obtained by medical students as a common and pervasive problem. Furthermore, uncertainty remains amongst students and clinicians as to who holds the responsibility of informing the patient of student involvement, which is frequently implicated in the disregarding of patient autonomy [9]. Adequate informed consent procedures are eroded by high patient volumes, lack of clarity concerning who is responsible for obtaining patient consent, and the prioritising of following instructions rather than protecting patient autonomy [8]. These factors are aggravated by unclear, absent, or de facto poorly implemented university informed consent guidelines concerning the involvement of medical students in patient care [7].

As per the Health Professions Council of South Africa (HPCSA) regulations, a patient has the right to be informed of medical student involvement in their care, and the skill level and qualifications of this student, and van Niekerk et al. recommend that students should state their academic status during interactions with patients [8]. A study in South Africa identified the lack of clear guidelines regarding patients’ rights in relation to medical student involvement in patient care [10].

### Patient confidentiality

Many patients in the literature express concern for the protection of their privacy [4, 7]. This concern extended to what their medical records should contain and who should have access to their records [11]. More patients expressed concern over the presence of medical students during consultations involving sensitive details [11].

### Patient perceptions

Factors influencing patient perceptions of medical students include ethnicity and age of the patient, their previous experience with medical students, the body part of the patient being examined, and the gender of the student [12]. It has been noted that patient’s acceptance of medical student participation greatly varies between studies and is particularly dependent on the type of participation [13]. For example, Graber et al*.* [14] found that a significant proportion of patients would refuse invasive procedures from students, while Santen et al*.* [15] found that most patients would allow medical students to perform minor procedures such as taking a history or physical examination. The level of student involvement expected by patients that refuse involvement was higher than expected by patients that were willing to participate [16]. Measures to inform patients prior to their involvement in medical training, i.e., providing them with formal notification and a clear role, lead to no substantial loss in patient involvement [9]. Patients have been shown to be more comfortable with prior notification [17]. There is a lack of consensus regarding the effect that certain factors have on the patient’s perception of students. Wright found that patients who had previously consulted with a student were more reluctant to have a student present during the consultation [18]. Conversely, Choundhury et al*.* found that such patients with previous experience of medical students tended to have a positive attitude towards student involvement in their care [12]. There is consensus that female patients, especially in areas concerned with reproductive health, are more likely to refuse student participation, particularly if the student is male [18–20]. Overall, the more positive the patient’s view concerning the importance of medical education, the more likely they are to agree to student participation and have an improved experience with the students [21].

There is conflicting and Western-centric research on patients’ views on their role in undergraduate medical education. Hence, there is a need to research this topic in a South African healthcare setting.

Consequently, this study aimed to identify and evaluate the factors that influence the willingness of patients to participate in undergraduate medical education, with the research question: “what factors influence a patient’s decision to participate in the clinical education of undergraduate medical students in public and private hospitals in South Africa?” In addition, this study further sought to establish patient views on their role in medical education – in both the public and private health sectors in selected Johannesburg hospitals – to aid in making recommendations on how clinical education may be modified to suit the best interests of students, patients, and practitioners.

## Methods

### Study design

A cross-sectional study was conducted using an anonymous, self-designed, English, 23-question paper questionnaire.

### Participants

The study participants were patients (aged ≥ 18 years) who were admitted to the medical, surgical, or gynaecological wards at the selected public academic and private hospital sites in Johannesburg, South Africa. Patients were approached by members of the research team to participate in the study – on a voluntary basis – by completing a questionnaire regarding their perceptions. All patients provided both verbal and written consent prior to participation, for which no compensation was provided. Paediatric patients, patients with an altered mental capacity, patients who were severely ill, non-English speaking patients and patients with an inability to provide informed consent were excluded from study participation.

Initially, a target sample size of 377 was generated based on an arbitrarily large number of participants (50,000) using Raosoft (Raosoft, Inc., 2004). This was likely a substantial overestimate of the total number of beds available in the specified departments at the respective study sites but was used as an estimate based on the lack of accurate information regarding patient numbers during the COVID-19 pandemic.

### Questionnaire

Prospective participants were requested to voluntarily complete a questionnaire regarding their perception of medical students in their care. As the research team did not identify any pre-existing questionnaires suited to the South African context, a self-designed questionnaire was used for this study. The developed questionnaire aimed to identify which factors were most influential when patients opted to partake in undergraduate medical education. It collected basic demographic information from patients and included multiple thematic sections namely an introduction (four questions including questions on previous exposure to medical students and willingness to participate in undergraduate medical education), consent (nine questions including specified student-patient interactions), confidentiality (two questions), ethics (3 questions), and, patient preferences and perceptions (5 questions including questions on title preferences, preferred level of seniority of students and consent to specific procedures and examinations conducted by students with an open-ended optional comments section). For the purpose of this study a procedure was defined as a task that was permitted to be performed by medical students in the South African context – a common example includes obtaining intravenous access. Each section consisted of a variety of question types including Lickert scale, multiple selection, and polar questions together with an optional open-ended comments section in which participants were given an opportunity to provide further insight on their perceptions. The questionnaire is available in Additional file 1.

### Data collection

A pilot study, involving 13 patients, to evaluate the effectiveness of the questionnaire as a research tool in the South African context was carried out at another public academic hospital in March 2021. Following this process, minor amendments were made to increase validity, reduce ambiguity, and elucidate some of the questions that were identified as problematic.

The revised questionnaire was then distributed in July and October 2021 in the Departments of Medicine, Surgery, and Gynaecology, at the selected public and private hospital sites. These departments were selected due to their ability to provide a broad selection of hospitalised adults in South Africa, with a diverse range of inpatient experiences. Owing to COVID-19 restrictions, which limited patient numbers and ward access during data collection, voluntary sampling was used for this study and a total of 201 completed questions – comprising of 150 from the public sector and 51 from the private sector – were received. These we retrieved by the research team and stored for analysis.

Ethics approval for this study was granted by the University of the Witwatersrand Human Research Ethics Committee (Medical) (Study no. M200865), and verbal and written informed consent was obtained from all study participants prior to enrolment.

### Data analysis

Following collection, the data was manually entered into an Excel spreadsheet and subsequently analysed in Python 3.9 (Anaconda Inc., Berlin, Germany) using the SciPy library and graphed with the Seaborn library. Descriptive statistics were performed on the responses. The principal statistical tests employed were Pearson’s Chi-squared test for independence (bidirectional one-tailed) and Fisher’s Exact test (two-tailed) when the sample size of the response was low. The Wilson Score Interval was used to calculate a 95% confidence interval. For all tests, *p* < 0,05 was regarded as significant. Missing data fields for individual questions were ignored as these made up a minute proportion of the results. Grossly inadequately completed questionnaires and questionnaires where age was not provided were excluded from the analysis. Qualitative data from the comments section were analysed using Braun and Clarke 6 step analysis [22]. After familiarising ourselves with data, initial codes were generated, followed by searching for common themes. These themes were reviewed, defined, and named [22].

## Results

There were 201 questionnaires collected. In the public sector sampling, 50, 51 and 50 questionnaires were collected from the departments of medicine, surgery, and gynaecology respectively. A similar departmental distribution was noted in the private sector with 17, 13 and 18 questionnaires collected from the departments of medicine, surgery, and gynaecology respectively, and the remaining 2 questionnaires were from an unknown department. A total of 138 (68,7%) female patients participated whilst the remaining 63 (31,3%) patients were male. The notable female preponderance can be attributed to sampling in the gynaecology departments. The mean age of participants was 40 years with a range of 72 years. The median length of hospital stay at the time of sampling was five days. In addition, 112 (55,7%) patients indicated a previous encounter with a medical student in their care (public: 66,0%; private: 25,5%; *p* < 0,001), of which 4 (3,6%) reported their previous experience with a medical student as ‘bad’ (public: 4,0%; private: 0,0%) (See Table 2).

### Willingness to participate in undergraduate medical education

The results of the study demonstrated that 169 (84,1%) hospital inpatients were willing to participate in undergraduate medical education. This comprised 125 (86,2%) patients in the public sector as opposed to 40 (78,4%) of patients in the private sector (*p* = 0,28). One hundred and thirty-seven (68,2%) participants across both sectors indicated that they would be willing to participate at any time with 78,7% of participants in the public sector and 37,3% of patients in the private sector reporting this (*p* < 0,001). In addition, 185 (93,0%) patients across both sectors indicated that they would be willing to allow a medical student to observe their interactions with a healthcare provider (public: 91, 9%; private: 96,1%; *p* = 0,53) whilst 180 (91,8%) patients indicated they would allow a medical student to participate in a consultation (public: 93,8%; private: 86,3%; *p* = 0,13) (See Table 2).

### Consent

In reference to informed consent, 130 (66,3%) participants across both sectors expressed that they believed they could withhold consent if they did not wish to participate in undergraduate medical education at any given time. This response was indicated by 60,3% of patients and 84,0% of patients in the public and private sectors, respectively (*p* = 0,004). In addition, 108 (54,0%) participants indicated that a verbal consent process would be satisfactory whilst the remaining 92 (46%) participants indicated that they would prefer a process of written consent. Variability across the two sectors was noted with 63 (42,3%) patients in the public sector indicating a preference to written consent when compared to 29 (47,5%) patients in the private sector (*p* = 0,59). Furthermore, 77 participants (39,1%) indicated a preference to a consent process prior to presentation for consultation whilst 120 (59,7%) indicated that consent sought at the beginning of the consultation would be sufficient (public: 52, 33,3%; private 24, 47,1%); *p* = 0,23) (See Table 2). Moreover, whilst the provision of consent remained similar when patients encountered medical students of the opposite gender, the provision of consent varied across procedures and willingness to consent to participation declined with the perceived degree of invasiveness of procedures as demonstrated in Table 1.

**Table 1:** Patient preferences for engagement with undergraduate medical students

	Total number that would permit procedure, setting, or examination, n (%)	Public, n (%)	95% Confidence Interval	Private, n (%)	95% Confidence Interval	*P* value
**Settings where patients would participate in undergraduate medical education**						
Anytime	137 (68,2)	78,7	71,4; 84,5	37,3	25,3; 51,0	0,001
Academic hospital	92 (45,8)	44,7	36,9; 52,7	49	35,9; 62,3	0,63
Same gender student	66 (32,8)	39,3	31,9; 47,3	13,7	6,9; 25,7	0,001
Professional present	141 (70,1)	66	58,1; 73,1	82,4	69,7; 90,4	0,05
Experienced student	93 (46,3)	48	40,2; 55,9	41,2	28,7; 54,8	0,42
More than 5 students	43 (21,4)	26,7	20,2; 34,3	5,9	2,0; 15,9	0,05
Never	6 (3,0)	2	0,7; 5,7	5,9	2,0; 15,9	0,17
**Procedures participants would permit to be performed on them by a medical student**						
Hx with professional	185 (92,0)	90	84,2; 93,8	98	89,7; 99,7	0,08
Hx with no professional	120 (60,0)	67,8	59,9; 74,8	37,3	25,3; 51,0	0,001
Reading file	164 (81,6)	81,3	74,3; 86,8	82,4	69,7; 90,4	1
MSK exam	163 (81,1)	80,7	73,6; 86,2	82,4	69,7; 90,4	1
Abdo. exam	158 (78,6)	78,7	71,4; 84,5	78,4	65,4; 87,5	1
CVS/Resp. exam	170 (84,6)	84,7	78,0; 89,6	84,3	72,0; 91,8	1
Venepuncture	140 (69,6)	76	68,6; 82,1	54,9	41,4; 67,7	0,05
IV Cannulation	142 (70,6)	74	66,4; 80,4	56,9	43,3; 69,5	0,05
Rectal examination	93 (46,2)	52,7	44,7; 60,5	27,5	17,1; 40,9	0,001
None	4 (0,02)	2	0,7; 5,7	2	0,3; 10,3	1
**Procedures participants would permit to be performed on them by a medical student of another gender**						
History with professional	185 (92,5)	92,6	87,3; 95,8	92,2	81,5; 96,9	1
History with no professional	122 (61,0)	69,1	61,3; 76,0	37,3	25,3; 51,0	0,001
Reading file	166 (83,0)	83,2	76,4; 88,4	82,4	69,7; 90,4	1
History	129 (79,5)	79,2	72,0; 84,9	80,4	67,5; 89,0	1
MSK exam	162 (81,0)	82,6	75,7; 87,8	76,5	63,2; 86,0	0,41
Abdo. exam	151 (75,5)	77,2	69,8; 83,2	70,6	57,0; 81,3	0,35
CVS/Resp. exam	157 (78,5)	79,2	72,0; 84,9	76,5	63,2; 86,0	0,7
Venepuncture	133 (66,5)	71,1	63,4; 77,8	52,9	39,5; 65,9	0,27
IV cannulation	133 (66,5)	70,4	62,7; 77,2	54,9	41,4; 67,7	0,06
Rectal/genital examination	86 (43,0)	52,7	41,1; 56,9	25,5	15,5; 38,9	0,001
None	9 (4,5)	3,4	1,4; 7,6	7,8	3,1; 18,5	0,24

### Confidentiality

Regarding confidentiality, 25 (14,4%) patients indicated that they believed that the presence of medical students in their consultation would invade their privacy. This was more prevalent in the public sector sampling (15,5%) when compared to the private sector sampling (3,9%) (*p* < 0,05) (See Table 2).

**Table 2:** Factors influencing engagement with undergraduate medical students

Question	Total no. of ‘yes’ answers, n (%)	Public, n (%)	Private, n (%)	*P* value
**Introductory questions**				
Previous exposure to medical student	112 (55,7)	99 (66,0)	13 (25,5)	< 0,0001
‘Good’ previous experience	98 (87,5)	95 (98,9)	13 (100)	1,0
Undergraduate medical education rated as ‘very important’	170 (86,3)	125 (85,0)	45 (90,0)	0,32
‘Comfortable’ to participate in undergraduate medical education	165 (84,2)	125 ( 86,2)	40 (78,4)	0,28
**Consent**				
Been asked about willingness to participate in undergrad. medical education	83 (41,5)	59 (39,6)	24 (47,1)	0,44
Allow to say ‘no’	130 (66,3)	88 (60,3)	42 (84,0)	0,004
Procedure with no consent	25 (12,6)	23 (15,5)	2 (3,9)	0,03
Allow student to observe	185 (93,0)	136(91,0)	49 (96,1)	0,49
Allow to participate	180 (91,8)	136(93,8)	44 (86,3)	0,17
Verbal consent (vs written)	108 (54,0)	86 (57,7)	22 (43,1)	0,10
Consent at beginning (vs prior to consultation)	120 (60,9)	93 (63,7)	24 (47,1)	0,23
**Confidentiality questions**				
Privacy violated by medical student presence	155 (77,5)	40 (36,7)	5 (9,8)	0,02
‘Very comfortable’ with medical student access to records	121 (60,2)	100 (66,7)	21 (41,2)	0,01
**Ethics**				
‘Duty to teach’: agree and strongly agree	127 (63,8)	106 (71,6)	21 (41,2)	< 0,001
‘Expect to participate’: agree and strongly agree	127 (63,8)	102 (68,9)	25 (49,0)	0,01
Ethical to train on real patients: agree and strongly agree	148 (74,4)	109 (73,6)	39 (76,5)	0,69

### Ethics

In terms of ethical considerations, across both sectors, 127 (63,8%) patients indicated ‘agreement’ or ‘strong agreement’ with the statement that they had a ‘duty to teach’ (public: 71,6%; private 41,2%; *p* < 0,001), whilst 33 (16,6%) patients indicated ‘disagreement’ or ‘strong disagreement’ with the same statement (public: 12,8%; private 27,5%; *p* < 0,001). In addition, 127 (63,8%) patients ‘agreed’ or ‘strongly agreed’ with a statement that ‘they should expect to participate in teaching whilst in hospital’ (public: 68,9%; private 49,0%; *p* < 0,05), whilst 30 (15,1%) patients ‘disagreed’ or ‘strongly disagreed’ with the same statement (public: 12,2%; private 23,5%; *p* = 0,05). Furthermore, 148 (74,4%) patients ‘agreed’ or ‘strongly agreed’ that it is ‘ethical’ to involve medical students in the care of ‘real patients’ (public: 73,6%; private 76,5%) whilst 15 (7,5%) patients indicated ‘disagreement’ or ‘strong disagreement’ with this statement (public: 9,5%; private: 2,0%; *p* = 0,08). These are further demonstrated in Table 2.

### Perceptions and preferences

In respect of patient perceptions, 170 (86,3%) patients rated the clinical training of medical students as ‘very important’ (public: 85%; private: 90%, *p* = 0,14), whilst 5 (2,5%) patients regarded the clinical training of medical students as ‘not important’ (public: 2%; private: 4%; *p* = 0,60). Patients also responded differently with regards to their willingness to undergo various procedures and examinations conducted by students of the same and opposite sex (See Table 1).

**Fig. 1:**
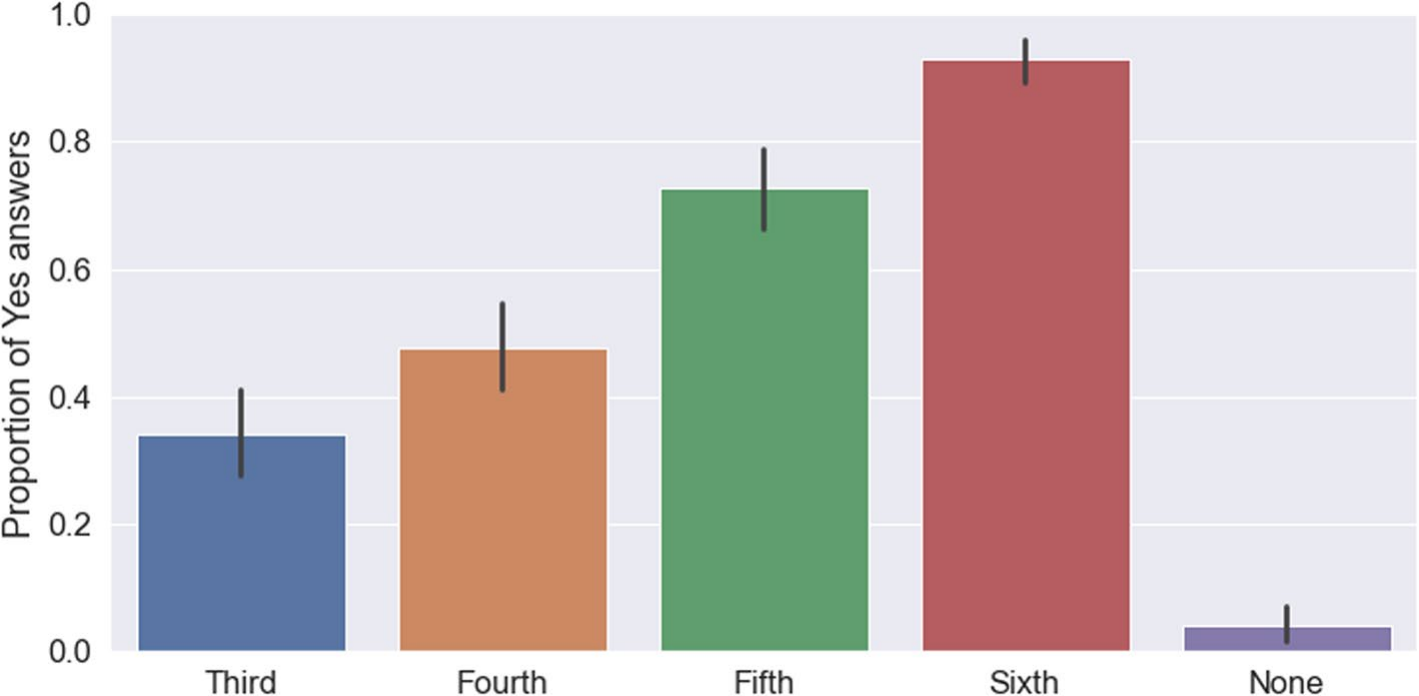
The proportion of patients willing to be seen by various years of medical student. (Black lines show confidence interval)

**Fig. 2:**
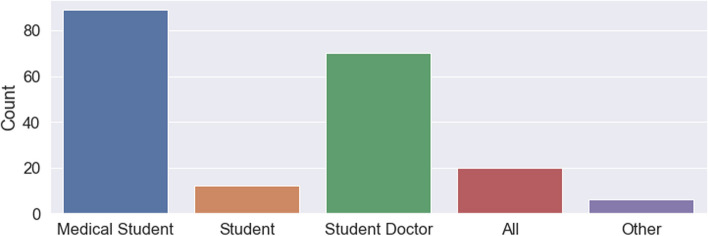
Preferred titles by which students should be introduced

Patients also indicated various reasons for participation in medical education including the beliefs that ‘students need to learn’ (public: 91%; private: 98%; *p* < 0,05), they can learn about their illness (public: 61,2%; private: 49,0%; *p* < 0,05), they can learn from students (public: 53,7%; private: 37,3%; *p* < 0,05), they might get better treatment (public: 39,3%; private: 17,6%; *p* < 0,001) and enjoyment (public: 35,8%; private: 17,6%; *p* < 0,05). These reasons are shown in Table 3. Despite this, 62 (31,5%) patients believed that the presence of a medical student would impede on the ability of healthcare professionals to manage their medical problems safely and effectively (public: 39,0%; private: 9,8%; *p* < 0,001). In terms of patient preferences, 185 (93,0%) patients would consent to the involvement of a final year medical student in their care (public: 91,9%; private: 96,1%; *p* = 0,53) whilst only 68 (34,2%) patients would consent to similar involvement by a third-year medical student (public: 39,2%; private: 19,6%; *p* = 0,01). These results are detailed further in the Fig. 1. In addition, patients indicated their main preferences on how students conducting themselves in the clinical space should be introduced to be ‘medical student’ and ‘student doctor’. These results are shown in the Fig. 2.

**Table 3:** Patient reasons for participation in undergraduate medical education

	**Total no., n (mean %)**	**Public, n (%)**	**95% Confidence Interval**	**Private, n (%)**	**95% Confidence Interval**	***P* value**
Students need to learn	183 (91,0)	88,7	82,6; 92,8	98	89,7; 99,7	0,05
To give back to community etc	143 (71,1)	72,7	65,0; 79,2	66,7	53,0; 78,0	0,47
To learn about illness	123 (61,2)	65,3	57,4; 72,5	49	35,9; 62,3	0,05
Learn from students	108 (53,7)	59,3	51,3; 66,9	37,3	25,3; 51,0	0,05
Better treatment	79 (39,3)	46,7	38,9; 54,6	17,6	9,6; 30,3	0,001
Enjoyment	72 (35,8)	42	34,4; 50,0	17,6	9,6; 30,3	0,05
Trained this way	33 (16,4)	16,7	11,6; 23,4	15,7	8,2; 28,0	1
None	7 (3,5)	4	1,8; 8,5	2	0,3; 10,3	0,68

Two major themes were found in the qualitative data, namely patients’ satisfaction and patients’ apprehensiveness. Patients’ satisfaction: patients felt the gratification of contributing to the training of future medical doctors which would in turn benefit the country. Patients felt that the presence of medical students afforded an opportunity to ask questions without fear and being heard. Patients’ apprehensiveness: patients suggested that students should not examine very ill patients or those experiencing too intense pain. Patients expressed being overwhelmed by the presence of too many students during ward rounds. They also raised concerns about consultants mentioning their diagnosis loud enough for other patients to hear, while teaching medical students.

## Discussion

To the best of our knowledge, this is one of a limited number of studies that compare and contrast patients’ perceptions across the public and private sectors in South Africa. This study has five principal findings aligned with its objectives: (1) the majority (84,2%) of hospital inpatients across both the public and private sectors are willing to participate in undergraduate medical education (2) the main factors which influenced this willingness included (a) the presence of a supervising professional (b) the degree of invasiveness of a procedure (c) the perceived expertise of the student (3) consistent with existing literature, most patients (87,58%) are willing to participate in undergraduate medical education as they believe that it enables the development of competent healthcare professionals (4) a significant proportion (66,3%) of patients do not feel compelled to participate in undergraduate medical education at any given time (5) variability in these findings exists across the public and private sectors.

It is noteworthy that 130 (66,3%) patients across both sectors felt that they could withhold consent should they not wish to participate in undergraduate medical education. Whilst consistent with existing literature by Menzes et al*.* in which it was noted that approximately 50% of participating patients, surveyed in a South African public hospital, felt they had the right to refuse interaction with students [23]. This is in contrast to existing literature by Rockey et al*.* in which it was found that almost 100% of patients surveyed in a United States hospital did not feel pressured to participate in teaching sessions [24]. This elucidates that although there is some degree of respect for patient autonomy as well as the presence of informed consent procedures in South African clinical environments, it is likely that this is insufficient when compared to resource riche settings. Menzes et al. further noted that it is often presumed that patients should participate in clinical teaching as they are benefitting from the care provided by doctors and other healthcare professionals at academic healthcare institutions [23]. However, patients have the right to both informed consent and autonomy. Yet this study by Menzes et al*.* study demonstrated that one-third of patients surveyed did not understand they were likely to encounter students [23]. In addition, the notable difference between the two sectors demonstrates the likelihood that such informed consent procedures and respect for autonomy are less prevalent within the public sector South African hospitals when compared to the private sector South African hospitals This can likely be attributed to patients in private sector hospitals having reduced contact time with students when compared to patients in public sector academic hospitals who may have daily contact time with students and have come to accept this as the norm. Furthermore, existing literature, by Maseko and Harris, noted that 16,8% of patients surveyed in a public health institution in South Africa rated their experiences as poorly patient-centred [25]. This is in contrast to just 3,2% of patients surveyed in a private health institution in South Africa who offered the same experience rating. Moreover, a study conducted by Ewunetu et al*.* in Ethiopia a low-middle income country with co-existing public and private health sectors, like South Africa had similar findings in which 66,3% of patients surveyed in the public health sector reported poor patient-centred care [26]. A similar setting there are similar challenges with regards to patient autonomy and informed consent in lower income public healthcare facilities [26]. Patients encountered in private sector institutions in South Africa are incurring additional personal expense for their care and hence are likely to have higher expectations for the quality of care they receive.

Pertaining to informed consent: 25 (12,6%) patients reported that procedures had previously been performed on them by medical students without prior consent (public: 15,5%; private: 3,9%; *p* < 0,05), whilst a further 4 (3,6%) patients from the public sector reported previous ‘bad’ experiences with medical students in their care. Informed consent is an essential component of clinical consultations which is taught in the early years of medical training [23]. These situations in which informed consent was not obtained by students, highlight the need for improvement and streamlining of informed consent procedures when medical students conduct themselves in the clinical environment. This notion is further elucidated in the study by Menzes et al*.* [23] who noted that “only 2% of the students surveyed in the study felt that the process of informed consent was necessary” with one third of the students surveyed reporting that they felt there was “no need for consent when undertaking physical examination or performing a procedure” [23].

This study assists in narrowing the literature gap on this topic by identifying reasons for patient participation in undergraduate medical education as well as identifying factors that facilitate or impede on their willingness to participate. In this study, the most common reasons for participation were identified as (1) assisting students in their learning (2) giving back to the community (3) learning more about their illness. These findings were consistent with existing literature by Dijk, Duijzer and Wienold in which it was noted that: “patients described a strong sense of having a meaningful contribution and personal fulfilment, because they were teaching patient-centredness, offering their body and authenticity, bolstering students’ confidence, fulfilling their responsibility to the broader community and improving the healthcare system” [27]. These reasons were further demonstrated in the open-ended comments section where patients expressed that they felt that “the learning process involves practice and theory, in order for students to learn correctly they must be given a guided opportunity of practising the theory learned”. A few other comments included patients expressing that they felt: “more comfortable talking to students and asking questions”, “students will become better doctors”, and “students will boost their confidence by being paired with the experienced”. The data and comments demonstrate that patients are aware of the value of their participation in undergraduate medical education as ‘real-life’ examples of what students learn from textbooks. In addition, patients also alluded to the role of medical students in helping future patients with comments such as “students must learn as they will be the doctors of tomorrow”. This implies that through their role in assisting students with their learning, they hope to improve the lives of future patients. This probably enforces the widely held belief that patients have an active role as teachers as opposed to being mere passive aids to learning [24].

In contrast to the belief that they could withhold consent and the desire to participate for the benefit of students learning which demonstrates the altruistic nature of many participants of the study, 127 (63,8%) patients indicated that they felt they had a ‘duty to teach’ (public: 71,6%; private 41,2%; *p* < 0,001) and that they should ‘expect to participate in teaching whilst in hospital’ (public: 68,9%; private 49,0%; *p* < 0,05) [20]. This demonstrates that patients do experience an extent of obligation in their suffering [21]. As such more concerted efforts need to be made to ensure that the participation of patients in undergraduate medical education does not induce any further physical or mental harm to the patient. This is particularly relevant in the public sector in which a notably larger proportion of patients – when compared to the private sector – expressed this obligatory view. In addition, standard guidelines, protocols, and procedures should be implemented across both sectors to ensure that patients understand the right to autonomy when opting to participate as well as the extent of their expected involvement prior to participating.

Furthermore, this study demonstrated variability in the willingness of patients to participate in undergraduate medical education with different clinical situations or procedures. Consistent with existing literature by Vaughan et al*.* [11], the results reflected a decreased willingness with increased perceived invasiveness of clinical procedures such as venepuncture, intravenous cannulation, and rectal examinations [11] (See Table 1). In addition, the results also documented a decreased willingness of patients to participate based on their perception of the student’s expertise in various years of study. One hundred and eighty-five (93,0%) patients would consent to the involvement of a final year medical student in their care (public: 91,9%; private: 96,1%; *p* = 0,53) whilst only 68 (34,2%) patients would consent to similar involvement by a third-year medical student (public: 39,2%; private: 19,6%; *p* = 0,01). The difference noted across the sectors in the willingness to participate in the presence of a third-year medical student can likely be attributed to the repeated encounters patients in the public sector have with medical students as well as their lack of awareness of the level of medical education these students have obtained at the time of the encounter. These situations once again highlight the importance of appropriately obtaining informed consent and an unambiguous explanation of the role of students [28].

The strengths of this study lie in its concentration on patient perceptions and thxe use of a pilot study to optimise the data collection tool to enhance validity and reliability of the data collected. In addition, the brief and concise nature of the questionnaire ensured that patients were able to complete the entire questionnaire and minimal data was incomplete.

Despite many strengths this study also has several limitations including a smaller sample size in the private sector (51 participants) when compared to the public sector (150 participants). The calculation of a sample size that was not based on the COVID-19 clinical setting in terms of the respective number of beds and number of patients may have impeded on the data collection. Some of the questions on the questionnaire may have not been interpreted as intended, thus leading to erroneous results. In addition, reliance on voluntary participation by patients meant that similarly to existing studies the questionnaire was likely to be completed by patients who were willing to participate in undergraduate medical education. Furthermore, limiting the study to English-speakers only does not accurately reflect the diversity present in South African hospitals whilst varying literacy levels may have hampered the ability of patients to accurately complete the questionnaire. Moreover, bias may have been introduced by the presence of medical students whilst patients completed the questionnaires. Despite these limitations, the results are generalisable to the broader South African context due to the similar structures of health facilities in both the public and private sectors as well as the presenting populations to such health facilities. Hence this study can facilitate the development of guidelines to streamline medical student patient interactions within the South African clinical space. This can subsequently lay a foundation for the development of similar guidelines in other low-middle income countries which have health care systems structured as that of South Africa.

As this study has demonstrated that patients in both the public and private sectors are willing to participate in undergraduate medical education, future research should aim towards understanding the perceptions of practitioners – with regards to teaching undergraduate medical students—in each of these sectors. Moving forward, patients should not be obligated to participate in undergraduate medical education and irrespective of the health sector their autonomy should always be respected. In addition, quality informed consent measures should be instituted for these practices and medical schools should institute practices that train medical students to introduce themselves unambiguously when conducting themselves in a clinical environment.

## Conclusion and recommendations

In conclusion, this study notes that there is no difference between the South African public and private sectors with regard to the willingness of patients to participate in undergraduate medical education. Consistent with existing literature, most patients are willing to participate as they believe that students need exposure to the clinical environment to facilitate adequate, appropriate learning which will foster their development as healthcare professionals. It also revealed that most patients have a positive experience. However, this study demonstrated that patients do not receive adequate forms of informed consent. As a result, more robust measures of informed consent need to be instituted in order to optimise the current role of the South African public health sector whilst facilitating the development of a similar role for the South African private health sector in undergraduate medical education in the future.

### Supplementary Information

**Additional file 1.**

## Data Availability

The datasets generated and/or analysed during the current study are not publicly available due to the data being potentially sensitive and not having formal ethics approval for sharing of the materials but are available from the corresponding author on reasonable request.
