# Supplementary material for: A cross-sectional study on factors influencing patient participation in undergraduate medical education in a public and private hospital in Johannesburg, South Africa
Source: BMC Med Educ. 2023 Sep 21;23:687. doi: 10.1186/s12909-023-04663-w (PMC10514977; doi:10.1186/s12909-023-04663-w)
Supplement: Supplementary file 1 — Additional file 1. [file 12909_2023_4663_MOESM1_ESM.pdf]

FACTORS INFLUENCING THE WILLINGNESS OF PATIENTS TO PARTICIPATE IN THE CLINICAL EDUCATION OF UNDERGRDUATE MEDICAL STUDENTS

Please provide us with some additional information about yourself.

Age: ________________ Sex: ________________ Duration of Hospital Stay: ________________

1. Introduction

a. Have you previously encountered the presence of a medical student in a consultation with your health care provider?

◯ Yes ◯ No

b. If you answered yes to the previous question (a), how would you best describe your experience with the presence of the medical student in your consultation?

◯ Good ◯ Bad ◯ Not Applicable

c. How would you rate the importance of practical clinical training of medical students?

◯ Very Important ◯ Moderately Important ◯ Not important

d. Would you feel comfortable if asked to participate in undergraduate medical student clinical education?

◯ Yes ◯ No

2. Consent

a. Have you been asked about your willingness to participate in clinical education?

◯ Yes ◯ No

b. When asked to participate in clinical education of undergraduate medical students, do you feel that you are allowed to say 'no' if you do not wish to participate?

◯ Yes ◯ Partly ◯ No

c. Has a procedure ever been performed on you by a medical student without prior consent?

◯ Yes ◯ No

d. If a medical student was present in your consultation with a health care provider would you allow them to observe the proceedings of the consultation?

◯ Yes ◯ No

e. If a medical student was present in your consultation with a health care provider would you allow them to participate in proceedings of the consultation?

◯ Yes ◯ No

f. Would you consent to a medical student performing any of the following tasks? (mark all that apply)

◯ Talking to you about your medical problem in the presence of a supervising professional

◯ Talking to you about your medical problem in the absence of a supervising professional

◯ Reading the notes in your patient file

◯ Examining your joints

◯ Examining your abdomen

◯ Examining and listening to your chest

◯ Taking blood from you

◯ Setting up a drip for you

◯ Performing a rectal examination

◯ None of these

g. When would you consent to medical student involvement? (mark all that apply)

◯ Anytime, as long as it helps me.

◯ In an academic hospital setting.

◯ When the student is the same gender as me.

◯ When the student is working in the presence of a supervising professional.

◯ When the student is experienced.

◯ When there are more than five students present.

◯ None of these

h. In the event of medical student presence in your consultation and care, what is your preferred method to obtain consent?

◯ Written ◯ Verbal

i. In the event of medical student presence in your consultation and care, how well in advance would you prefer to be informed of their presence and involvement?

◯ Before arriving for my consultation ◯ At the beginning of my consultation

3. Confidentiality

a. Do you feel that your privacy is invaded by the presence of medical students in your consultation?

◯ Yes ◯ No

b. How comfortable are you with medical students having access to your medical records?

◯ Very comfortable ◯ Somewhat comfortable ◯ Uncomfortable

4. Ethics

a. Do you believe that patients have a 'duty to teach' when they are in the hospital?

◯ Strongly agree ◯ Agree ◯ Neutral ◯ Disagree ◯ Strongly disagree

b. Do you believe patients should expect to participate in medical student training when they are in hospital?

◯ Strongly agree ◯ Agree ◯ Neutral ◯ Disagree ◯ Strongly disagree

c. Do you believe it is ethical to allow medical students to be involved in the care of real patients?

◯ Strongly agree ◯ Agree ◯ Neutral ◯ Disagree ◯ Strongly disagree

5. Patient's Preferences and Perceptions

a. Do you believe that medical students' involvement in your consultation and care may prevent the health care provider from safely and effectively managing your health problem?

◯ Yes ◯ No

b. If a student is participating in your care, how would you prefer students to be introduced?

◯ Medical student
◯ Student doctor
◯ Student
◯ Other, please specify: ______________________________

c. Would you allow a student of the opposite gender to perform any of the following tasks? (mark all that apply)

◯ Talk to you about your medical problem in the presence of a supervising professional
◯ Talk to you about your medical problem in the absence of a supervising professional
◯ Read the notes in your patient file
◯ Take a medical history from you
◯ Examine your joints
◯ Examine your abdomen
◯ Examine and listening to your chest
◯ Take blood from you
◯ Set up a drip for you
◯ Performing a rectal or similarly invasive examination
◯ None of these

d. Would you allow the following levels of medical students be involved in your care…? (mark all that apply)

◯ Third year

◯ Fourth year

◯ Fifth year

◯ Sixth year

◯ None of these

e. What are the reasons that you would allow for the participation of medical students in your consultation and care?

◯ Students need to learn

◯ To give back to society/ the community/ the hospital/ the health care provider

◯ To learn more about your illness

◯ I can learn from the students

◯ I might get better treatment

◯ I enjoy it

◯ I was trained in this way

◯ I would not consent to medical student participation in my care.

6. Additional Comments

If you have an additional comments, please write them here.

____

____

____

____

____

____

Thank you for your time!
